# Prevalence of thyroid dysfunction in patients with diabetes mellitus

**DOI:** 10.1186/1758-5996-5-58

**Published:** 2013-10-09

**Authors:** Cátia Cristina Silva Sousa Vergara Palma, Marco Pavesi, Verônica Guedes Nogueira, Eliete Leão Silva Clemente, Maria de Fátima Bevilacqua Motta Pereira Vasconcellos, Luiz Carlos Pereira, Fernanda Faissol Pacheco, Tássia Gomide Braga, Ludmila de Faria Bello, Juliana Oliveira Soares, Stefanie Cathren Fenizola dos Santos, Vinícius Paes Leme Cavalcante Campos, Marília Brito Gomes

**Affiliations:** 1 Diabetes Unit, State University Hospital of Rio de Janeiro (UERJ), Avenida 28 de setembro, 77, Serviço de Diabetes, Terceiro andar, Vila Isabel, Rio de Janeiro, Brazil; 2 CLIF Data Centre, European Consortium on Liver Failure, Hospital Clínic de Barcelona, Barcelona, Espanha

**Keywords:** Thyroid dysfunction, Prevalence, Diabetes mellitus

## Abstract

**Background:**

Diabetes mellitus (DM) and thyroid dysfunction (TD) are the two most common endocrine disorders in clinical practice. The unrecognized TD may adversely affect the metabolic control and add more risk to an already predisposing scenario for cardiovascular diseases. The objective of this study was to investigate the prevalence of TD in patients with type 1 and type 2 diabetes mellitus (T1DM and T2DM).

**Methods:**

This is an observational cross-sectional study. Three hundred eighty-six (386) patients with T1DM or T2DM that regularly attended the outpatient clinic of the Diabetes unit, Hospital Universitário Pedro Ernesto, participated in the study. All patients underwent a clinical and laboratory evaluation. Thyroid dysfunction was classified as clinical hypothyroidism (C-Hypo) if TSH > 4.20 μUI/mL and FT4 < 0.93 ng/dL; Subclinical hypothyroidism (SC-Hypo) if TSH > 4.20 μUI/ml and FT4 ranged from 0.93 to 1.7 ng/dL; Subclinical hyperthyroidism (SC-Hyper) if TSH < 0.27 μUI/ml and FT4 in the normal range (0.93 and 1.7 ng/dL) and Clinical hyperthyroidism (C-Hyper) if TSH < 0.27 μUI/ml and FT4 > 1.7 μUI/mL. Autoimmunity were diagnosed when anti-TPO levels were greater than 34 IU/mL. The positive autoimmunity was not considered as a criterion of thyroid dysfunction.

**Results:**

The prevalence of TD in all diabetic patients was 14,7%. In patients who had not or denied prior TD the frequency of TD was 13%. The most frequently TD was subclinical hypothyroidism, in 13% of patients with T1DM and in 12% of patients with T2DM. The prevalence of anti-TPO antibodies was 10.8%. Forty-four (11.2%) new cases of TD were diagnosed during the clinical evaluation. The forty-nine patients with prior TD, 50% with T1DM and 76% with T2DM were with normal TSH levels.

**Conclusions:**

We conclude that screening for thyroid disease among patients with diabetes mellitus should be routinely performed considering the prevalence of new cases diagnosed and the possible aggravation the classical risk factors such as hypertension and dyslipidemia, arising from an undiagnosed thyroid dysfunction.

## Background

Diabetes Mellitus (DM) and thyroid dysfunction (TD) are the two most common endocrine disorders in clinical practice [1]. The association between DM and TD is widely known, with the first studies published in 1979 [2]. Since then, several studies in different countries were conducted to estimate the prevalence of TD in diabetic patients. There is great variability in the prevalence of TD in general population, ranging from 6.6% to 13.4% [3,4]. In diabetic patients, the prevalence is still greater and varies from 10 to 24% [4,5]. These differences can be explained by different diagnostic criteria of TD, the degree of iodine intake among different regions, different sensitivities of the TSH assays and the large population diversity [6]. The relationship between TD and DM is characterized by a complex interaction of interdependence. Screening of TD, especially the subclinical dysfunction, in patients with DM is justified because most patients can be asymptomatic. Determine the prevalence of clinical and subclinical thyroid disease in diabetic patients in our country and its implications in the course of diabetes and known factors for cardiovascular risk is necessary. The aim of this study is to investigate the prevalence of TD in patients with type 1 (T1DM) and type 2 diabetes mellitus (T2DM) in clinical routine.

## Methods

This is an observational cross-sectional study. Three hundred eighty-six (386) patients with T1DM or T2DM that regularly attended the out-patient clinic of the unit of Diabetes, at Hospital Universitário Pedro Ernesto participated in the study. The inclusion criteria was duration of DM longer than 6 months for patients with T2DM or 1 year for those with T1DM. Patients were diagnosed to have type 2 DM when it was diagnosed with an age ≥30 years, without insulin use in the first year after diagnosis and without history of ketosis or ketonuria. In T1DM patients, the diagnosis was based on typical clinical presentation, a variable degree of weight loss, polyuria, polydpsia and polyphagia and the need to use insulin continuously since the diagnosis without discontinuation and medical follow-up for at least 1 year. Written informed consent for the study was obtained from all of the patients aged 18 years or older or from the parents or guardians of the patients younger than 18 years. Patients or guardians who were unable to understand and sign the informed consent, pregnant women, patients with recent interventions: pulse corticosteroids and/or radioiodine, use of amiodarone and with a history of hospitalization for less than 6 months were excluded.

The study protocol was approved by the research ethics committee of Hospital Universitário Pedro Ernesto (HUPE).

All patients underwent a clinical and laboratory evaluation. The demographic data were obtained from a survey. The following variables were assessed: gender, age (years), ethnicity (Caucasian/non-caucasian), duration of DM (years), BMI, blood pressure (systolic and diastolic). Data on comorbidities such as hypertension, dyslipidemia, previous TD (hyperthyroidism, hypothyroidism, nodules, cancer), were also taken. Blood samples were obtained after a 12 hour overnight fast for biochemical analysis: fasting and 2 h-postprandial glycemia, HbA1c, anti-thyroperoxidase antibody (anti-TPO), free thyroxine (FT4) and thyrotropin (TSH). The reference values and intra-assay and inter-assay coefficients of variation were, respectively, <34 IU/mL, 3.4 and 7.6 for anti-TPO; 0.93 to 1.7 ng/dL, 1.8 and 3.0 for FT4; 0.27 to 4.20 μUI/ml, 1.9 and 2.2 for TSH.

Thyroid dysfunction was classified as clinical hypothyroidism (C-Hypo) if TSH levels were greater than 4.20 μUI/mL and FT4 levels were lower than 0.93 ng/dL; subclinical hypothyroidism (SC-Hypo) if TSH levels were greater than 4.20 μUI/ml and FT4 levels ranged from 0.93 μUI to 1.7 ng/dL, subclinical hyperthyroidism (SC-Hyper) if TSH levels were lower than 0.27 μUI/ml and FT4 levels ranged from 0.93 and 1.7 ng/dL and clinical hyperthyroidism (C-Hyper) if TSH levels were lower 0.27 μUI/ml and FT4 levels were higher than 1.7 ng / dL. Autoimmunity were diagnosed when anti-TPO levels were greater than 34 IU/mL. The positive autoimmunity was not considered as a criterion of thyroid dysfunction.

The sample size was calculated to estimate a prevalence of 10%. About 300 patients with T2DM and 80 patients with T1DM allowed to estimate the expected prevalences by means of 95% confidence interval with an absolute precision of ± 3.5% and ± 4,5%, respectively. Statistical analysis was performed using SPSS (*Statistical Package for the Social Sciences*) for Windows (version 15.0). Categorical variables were described as frequency (percentage), mean ± standard deviation were used for continuous parameters. Differences between two groups were compared by the Student T test. For non-parametric variables, the data are presented as median (min-max). In this case, the nonparametric Mann–Whitney test was used for statistical comparisons. Categorical variables were compared between two or more groups using the Chi-square test. For all analyses, a two-tailed p-value of <0.05 was considered statistically significant.

## Results

Overall 386 patients, 82 (21.2%) with T1DM and 304 (78.8%) with T2DM, were included. Clinical and demographic data of the studied population are presented in Table 1.

**Table 1:** Clinical and demographic data of the studied population

**Variable**	**T1DM**	**T2DM**
**N**	82	304
**Gender (female) n (%)**	43 (52%)	204 (67%)
**Age (years)**	33.5 ± 15,8	60.7 ± 10.6
**Diabetes duration (years)**	14.6 ± 11.7	14.8 ± 10.5
**Ethnicity, n (%)**		
**Caucasian**	37 (45%)	127 (42%)
**Non-caucasion**	45 (55%)	177 (58%)
**Hypertension (yes)**	16 (19.5%)	243 (79.9%)
**Dyslipidemia (yes)**	21 (25.6%)	209 (68.8%)
**Previous thyroid disease n(%)**		
**Yes**	12 (15%)	37 (12%)
**No**	61 (74%)	184 (60%)
**Unknown**	9 (11%)	83 (28%)
**BMI (kg/m**^**2**^**)**	24,4 ± 5,2	30,3 ± 5,1
**sBP (mmHg)**	111 ± 16	131 ± 20
**dBP (mmHg)**	79 ± 10	75 ± 11
**FPG (mg/dL)**	141 ± 85,5	139 ± 74,9
**2 h-PPG (mg/dL)**	216 ± 96,0	215 ± 81,5
**HbA1c (%)**	12,3 ± 3,1	8,3 ± 1,8
**TSH (μUI/mL)**	2,60 ± 1,80	2,53 ± 2,24
**FT4 (μUI/mL)**	1,29 ± 0,17	1,31 ± 0,33
**Anti-TPO (yes)**	12 (14.6%)	30 (9.9%)

Data are presented as mean ± SD, n (%). sBP, systolic blood pressure; dBP, diastolic blood pressure; BMI, body mass index; FPG, fasting blood glucose; 2 h- PPG, 2 h postprandial glucose.

The prevalence of TD in all diabetic patients was 14,7%. Autoimmunity, considered as anti-TPO positive, were present in 12 (14,6%) patients with T1DM and in 30 (9,9%) in T2DM patients.

The prevalence of SC-Hypo was 13% in T1DM patients without previous TD and was the only TD found in this group. In the group of patients with T2DM without previous TD, the prevalence of SC-Hypo was 12%, C- Hypo 0,7%, SC- Hyper 0,3% and C-Hyper 0,3%. Data shown in Table 2.

**Table 2:** The frequencies of thyroid dysfunction of T1DM and T2DM patients without prior TD

**Thyroid function**	**T2DM (n = 267)**	**T1DM (n = 70)**
**Euthyroidism**	232(86)	61(87)
**SC-Hypo**	31(12)	9(13)
**C-Hypo**	2(0,7)	0
**SC-Hyper**	1(0,3)	0
**C-Hyper**	1(0,3)	0

SC-Hypo,subclinical hypothyroidism, C-Hypo,clinical hypothyroidism, SC-Hyper,subclinical hyperthyroidism,C-Hyper, clinical hyperthyroidism. Data are presented as n (%).

Forty- four patients (13%) were diagnosed with TD during the clinical evaluation. We diagnosed 9 (13%) new cases of SC-Hypo in patients with T1DM and 31 (12%) new cases of SC-Hypo in patients with T2DM who denied or ignored prior TD. SC-Hypo was the most TD found in this diabetic group. Data presented in Table 2. In patients who reported previous TD, 28 (76%) of 37 T2DM patients and 6 (50%) of 12 T1DM patients had TSH and FT4 levels in the normal range. Data presented in Table 3.

**Table 3:** The frequencies of thyroid dysfunction of T1DM and T2DM patients with prior TD

**Thyroid function**	**T2DM (n = 37)**	**T1DM (n = 12)**
**Euthyroidism**	28(76)	6(50)
**SC- Hypo**	7(19)	5(42)
**C-Hypo**	0	0
**SC-Hyper**	2(5)	1(8)
**C-Hyper**	0	0

SC-Hypo,subclinical hypothyroidism, C-Hypo,clinical hypothyroidism, SC-Hyper,subclinical hyperthyroidism,C-Hyper, clinical hyperthyroidism. Data are presented as n (%).

## Discussion

This study demonstrated a 14,7% prevalence of TD in the diabetic patients studied. Subclinical hypothyroidism was the most frequent dysfunction found corresponding to 11,8% of the patients which was similar to studies already described in the literature [2,7] but higher than that reported in studies with non-diabetics [8-10].

Analyzing data from T1DM patients, the frequency of TD was 13% of SC -Hypo in patients without prior TD and 14,6% anti-TPO positive. In the study by *Ramos et al.*[11], where 126 patients with T1DM were evaluated, the prevalence of TD was 20.6%. However, they considered a positive test for autoimmunity as TD. In the same study, the prevalence of SC-Hypo was 13.5% and anti-TPO antibodies was 18.2%, but patients were younger compared to our study. In the study by *Souza et al.*[12], evaluating 101 patients with T1DM, the prevalence of Sc-Hypo was 6.5% but FT4 was not used for diagnosis. A prevalence of 30.7% of at least one antibody was found positive in the same study, where 3 antibodies were assayed: anti-thyroglobulin, anti-microssomal, and anti-TPO. The variability of the laboratorial methods on screening of thyroid autoimmunity could influence the results described above because the latest studies use anti-TPO as preferred test [12].

According to various studies, the prevalence of autoimmunity in T1DM patients may vary between 3 and 50% [13,14]. One factor that may account for this variability could be the different laboratory methods used for the evaluation of autoimmunity.

Nine new cases of DT were diagnosed in patients with T1DM, whom ignored or denied prior TD. Patients whom already known to have TD, only half (50%) presented hormone levels in normal range. This data are consistent with the NHANES study [10], where only 67% of individuals who self-reported having TD were adequately treated. Non-adherence to therapy can be explained by factors such as: the excess of medications used due to comorbidities, adverse effects of medications, high cost, education of patients that limits access to information, the asymptomatic nature of the disease(in some cases) in which the importance of drug treatment is not recognized [15]. Data from the study of “Adherence to Medication for Chronic Diseases (diabetes and hypertension) in the City of Teresina” demonstrated that the degree of compliance percentage was lower than the “recommended” [16] level of 80%. Other Brazilian studies demonstrated adherence rates of 11% in Bahia and 66.6% in São Paulo [17].

In the analysis of T2DM patients, we observed a frequency of all TD of 13,1% and SC-Hypo of 12%. This frequency found is higher than those described in the study of *Fremantle* (8.6%) [18] and in the study *Chu et cols*. (8.4%) [19]. Previous studies have shown that the risk of thyroid dysfunction increases with age [11,15]. It is important to emphasize that our patients with SC-Hypo are older than the two earlier studies afore mentioned. These findings may be more favorable to subclinical abnormalities that could explain the higher frequencies found in our study. Subclinical TD are characterized with biochemical changes without inclusion of clinical signs or symptoms [20].

We observed a overall frequency of 10,8% positive anti-TPO, 14,6% in T1DM and 9,9% in T2DM. The epidemiological study of Whickam [21], showed that 55% of patients with elevated TSH and positive anti-thyroid antibodies progressed to clinical hypothyroidism in contrast with 33% of those who had elevated TSH and negative antibodies.

Some limitations of our study must be discussed. This study was a cross- sectional with internal validity and we used a convenience sample of diabetic patients already treated in an university hospital. Some types of selection bias may have occurred because these patients are already under medical care. Some patients whom already had prior knowledge of their thyroid function could have some nonspecific symptoms of TD but not yet with laboratorial confirmation. They might be more interested on research. However, all patients were submitted to the same study protocol.

Other limitation of the study is that only one sample of TSH and FT4 was collected, which may have contributed to a high frequency of TD, since the phenomenon of regression to the mean was not minimized by a second sample. However, the dosage of FT4 is not always included in other prevalence studies like NHANES [10]. The level of FT4 add a greater specificity in the detection of TD.

The strength of this study is the great number of patients (n = 386), more than described in other Brazilian studies with diabetic patients mentioned previously and the dosage of TSH, FT4 and anti-TPO using ultra-sensitive laboratory tests performed in all patients.

This study joins previous studies to determine the prevalence of thyroid dysfunction in diabetic patients in Brazil and also includes a greater number of type 2 diabetic patients whose prevalence is poorly described in previous studies.

## Conclusion

In conclusion, the results of this cross-sectional study showed a high prevalence of TD in the diabetic population which indicates that screening for thyroid disease among patients with diabetes should be routinely performed. The prevalence of new cases of TD diagnosed and the possible aggravation of classical risk factors such as hypertension and dyslipidemia, arising from an undiagnosed thyroid dysfunction can lead to an increased cardiovascular risk in these patients. However, we reinforce that prospective studies and with more patients are necessary to clarify the impact of thyroid disease in diabetic patients.

## Abbreviations

C-Hypo: Clinical hypothyroidism; C-Hyper: Clinical hyperthyroidism; DM: Diabetes mellitus; SC- Hypo: Subclinical hypothyroidism; SC- Hyper: Subclinical hyperthyroidism; TD: Thyroid disfunction; T1DM: Type 1 diabetes mellitus; T2DM: Type 2 diabetes mellitus.

## Competing interests

The authors declared that they have no competing interests related to this manuscript.

## Authors’ contributions

CCSSVP: has written the manuscript and collected data. MP: did the statistical analysis. VGN, ELSC, MFBMPV, LCPJ, FFP, TGB, LFB, JOS, SCFS, VPLCC: have collected data. MBG: has corrected the manuscript. All authors read and approved this final manuscript.
